# Modelling the impact of mass drug administration with ivermectin or moxidectin on human onchocerciasis when there is sub-optimal response of parasites to treatment

**DOI:** 10.21203/rs.3.rs-10311498/v1

**Published:** 2026-07-20

**Authors:** Rebecca H. Chisholm, Shilian Xu, Kwadwo K Frempong, Himal Shrestha, Joseph Kwadwo Larbi Opare, Odame D. Asiedu, Ernest Mensah, Makedonka Mitreva, Warwick N Grant, Shannon M Hedtke

**Affiliations:** 1 Department of Mathematical and Physical Sciences, La Trobe University, Bundoora, Australia; 2 Melbourne School of Population and Global Health, The University of Melbourne, Melbourne, Australia; 3 Department of Microbiology, Anatomy, Physiology and Pharmacology, School of Agriculture, Biomedicine and Environment, La Trobe University, Bundoora, Australia; 4 Current address: Faculty of Biology, Technion-Israel Institute of Technology, Haifa, Israel; 5 Noguchi Memorial Institute for Medical Research, College of Health Sciences, University of Ghana, Accra, Ghana; 6 Department of Microbiology and Immunology, Microbiological Diagnostic Unit Public Health Laboratory, University of Melbourne, Melbourne, Victoria, Australia; 7 Neglected Tropical Diseases Program, Disease Control and Prevention Department, Public Health Division, Ghana Health Service, Accra, Ghana; 8 Department of Medicine, Washington University in St. Louis and McDonnell Genome Institute, St. Louis, Missouri 63108, USA; 9 La Trobe Institute for Molecular Science, La Trobe University, Bundoora, Victoria, Australia

**Keywords:** *Onchocerca volvulus*, moxidectin, ivermectin, sub-optimal response, modelling, treatment strategy, river blindness

## Abstract

Onchocerciasis is a disease caused by the filarial nematode parasite *Onchocerca volvulus* and is transmitted by blackflies after they ingest larval offspring, microfilariae, found in the skin of infected people. The elimination of transmission within endemic communities in sub-Saharan Africa, the Americas, and Yemen depends on mass drug administration of ivermectin (MDAi) at high treatment coverage until the prevalence of the parasite in people or in blackflies reaches below a threshold value. In Africa, there are a number of communities where worms that are less susceptible to the effects of ivermectin, i.e., that are sub-optimally responding (SOR), appear to be contributing to the persistence of onchocerciasis despite high-coverage MDAi. We have incorporated this variation in an epidemiological model to explore how the presence of SOR worms impacts the prevalence of people with skin microfilariae, fitting the model to epidemiological data from a community in Ghana, Asubende, where SOR has been detected. Incorporating a cumulative effect of ivermectin into the model fits the data better than if no cumulative effect is assumed. Simulations indicate that elimination is possible under biannual MDAi even if SOR worms are present in the starting population unless the baseline prevalence is hyperendemic. In a hyperendemic region, elimination is predicted if there is no SOR, but when SOR worms are included in the model, elimination is not reached even after twenty years of biannual MDAi with a treatment coverage of 80%. Biannual MDAi increases the proportion of reproducing female worms that would be characterized as SOR compared to annual MDAi. However, when using mass drug administration with moxidectin (MDAm), elimination becomes possible with fewer rounds of MDA even if there are worms that suboptimally respond to moxidectin. Our results support concerns raised in the research community that SOR to ivermectin in *O. volvulus* could result in persistent transmission despite high-coverage, biannual MDAi, but that MDAm could pose a solution, should safety and efficacy trials support its distribution.

## Background

Macrocyclic lactones such as ivermectin or moxidectin continue to be a key weapon in the fight against nematode parasite infections. However, evidence is mounting that the efficacy of the drugs used against human or animal parasites varies significantly within and between populations, that this variation is a complex genetic trait, and that evolutionary selection increases the proportion of parasites with reduced drug efficacy when these drugs continue to be used over time (e.g., *Dirofilaria immitis*: [1], *Onchocerca volvulus*: [2], *Haemonchus contortus:* [3], *Teladorsagia circumcincta*: [4]). For elimination campaigns to be effective against human nematode parasites, epidemiological models used to assess alternative intervention strategies need to incorporate selection for decreased response to these drugs [5, 6]. While such a model has been derived for soil-transmitted helminths [7], models for nematodes transmitted by insect bites, such as the parasites that cause river blindness or lymphatic filariasis, are still lacking.

An estimated 246 million people are at risk of infection with the nematode parasite *O. volvulus*, which causes river blindness or onchocerciasis [8]. Pathologies of the disease include extreme itching, dermatitis, and depigmentation, irreversible vision loss leading to blindness, and neurological complications, including epilepsy and Nakalanga syndrome [9, 10]. These parasites are transmitted by blackflies in the genus *Simulium*: when a blackfly takes a blood meal from an infected person, it ingests the microscopic larvae, or microfilariae (mf), in that person’s skin. In the blackfly, larvae develop into stage 3 infective larvae (iL3), which enter the skin of a new person during a second blood meal. These larvae mature into fertile adult worms, which form “nests” within subcutaneous nodules in the person’s skin. Female worms tend to remain sessile within a nodule, while males traverse between nodules to mate [11, 12] and can inseminate multiple females, who in turn mate with multiple males [13]. A single inseminated female worm has the potential to produce a million mf over a year [14], and one person can be infected with as many as 4177 worms at a given time [11, 15]. Individual worm burdens are larger in areas where blackfly biting rates, and thus the likelihood of transmission, are higher [16].

The morbidity and associated economic impact of onchocerciasis has motivated large-scale elimination programs in sub-Saharan Africa, using mass drug administration of the macrocyclic lactone ivermectin (MDAi) to all members aged 5 years and older in at-risk communities [17, 18]. Ivermectin has at least two effects on *O. volvulus*: 1) an acute microfilaricidal effect that results in the rapid and almost complete removal of mf in the skin of treated hosts, and 2) an embryostatic effect on adult female worms that results in the temporary inhibition of the release of new mf [19]. Ivermectin thus acts by interrupting the transmission cycle, preventing new infections, rather than by killing adult worms. Because ivermectin does not kill adult *O. volvulus*, the World Health Organization (WHO) guidelines recommend that MDAi be continued annually for at least 12–15 years (i.e., for longer than a worm’s typical lifespan) given a therapeutic coverage of at least 80% [18].

A second macrocyclic lactone, moxidectin, has been approved for use in humans to treat onchocerciasis in people at least 12 years of age [20, 21, 22]. Clinical studies indicate that the microfilaricidal and embryostatic effects of moxidectin are both superior in their extent and duration compared to ivermectin [21, 22, 23, 24, 25]. As a result of safety and efficacy studies, including the safety of a single-dose in children aged four to eleven [26], Ghana has initiated the global first use of moxidectin for mass drug administration (MDAm) in target communities.

Because of its multi-host life cycle, with complex density-dependent interactions in each host, models of *O. volvulus* have continued to be adapted to improve their predictive utility. Both stochastic, individual-based models (ONCHOSIM [27] and EPIONCHO-IBM [28]) and deterministic, population-based models (EPIONCHO [29]) have been used to suggest optimal intervention strategies, including the number of rounds of ivermectin to reach elimination [30, 31], the effects of combining MDAi and other interventions [32, 33, 34], the impact of the geographic scale of intervention [35, 36], heterogeneity in individual exposure to transmission [37, 38], a cumulative effect of ivermectin on female fertility [39, 40], and the comparative efficacy of MDAi and MDAm for elimination [20, 41].

However, none of these epidemiological models have incorporated within-population variable parasite response to ivermectin, despite evidence that this is playing an increasingly important role in the ongoing persistence of onchocerciasis in many communities in sub-Saharan Africa. The “sub-optimal response” (SOR) of *O. volvulus* to ivermectin includes observations of return to fertility of individual adult female worms as soon as 60 days post-ivermectin exposure with an associated rapid return of mf in the skin [21, 22, 42, 43, 44, 45, 46, 47, 48, 49], as well as decreased efficacy of ivermectin in clearing mf [22, 23, 50]. SOR has been observed even in areas where there has been no history of ivermectin [22], and higher frequencies of SOR are identified in regions with longer histories of MDAi [2].

In this study, we adapt a deterministic *O. volvulus* transmission model to incorporate variable response to treatment (EPIONCHO [39, 51]). Our model accounts for two, heritable *O. volvulus* phenotypes with different responses to the embryostatic effect of ivermectin: female good-responding worms (GR) with a slow return to fertility and female SOR worms that recover fertility much faster following exposure to ivermectin. We parameterise our model to reflect *O. volvulus* transmission and MDAi frequency and coverage in one of the first communities where SOR was identified—Asubende, Ghana—which underwent a period of annual MDAi (from 1987–2006) and a subsequent period of biannual MDAi (from 2007–2024). We compare predictions from our model under this real scenario with those from an analogously parameterised model that does not account for variable parasite response to ivermectin. We then contrast the predictions of our model under different assumptions about the level of endemic transmission and frequency of SOR worms in the population prior to MDAi, the frequency and duration of MDAi, and under the theoretical impact of MDAm with superior microfilaricidal and embryostatic effects on both the GR and SOR phenotypes.

## Methods

### Mathematical model

**Figure 2.**
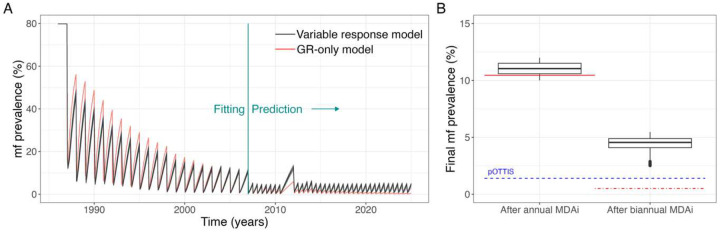
Predictions for mf prevalence following MDAi under the variable response model and the GR-only model calibrated to the Asubende data from 1987–2006. Red lines indicate results for GR-only model; black lines represent results under the variable response model. (A) Accepted trajectories of the mf prevalence (presented as the percentage of the population with positive mf load) over time measured in years following the start of the annual MDAi program in 1987 that switched to biannual rounds in 2006 (except for two skipped rounds during 2011). The vertical line is positioned at the timepoint where we fit the models to data after the annual MDAi program. Beyond this timepoint, the models are predicting the effects of biannual MDAi. (B) The boxplots summarise the distributions for the final mf prevalence under the variable response model, and the horizontal solid red lines show the predicted final mf prevalence under the GR-only model following (left) the 20-year annual MDAi program from endemic equilibrium in Asubende; and (right) the 18-year biannual MDAi program from the end of the 20-year annual MDAi program in Asubende. The horizontal dashed blue line is positioned where the mf prevalence equals pOTTIS.

The population-based deterministic model version of EPIONCHO proposed by Turner and colleagues [52] describes the transmission of *O. volvulus* and the effects of MDAi within a single population with age- and sex-structured rates of exposure of humans to vectors. Here, we adapt this model so that it incorporates variable responses of worms to treatment, under the assumption of homogenous rates of exposure to the vector. While the response of worms to macrocyclic lactones is likely to be a continuous phenotype, we modified this model assuming worms fall into two categorical treatment response phenotypes: SOR or GR. The model is shown schematically in Figure 2.

We formulate the model as a system of delay differential equations (Equations (1)–(4)) describing the rate of change with respect to time, *t*, of the mean number of fertile female adult worms of phenotype *i* per host (*F*_*i*_), the mean number of non-fertile female adult worms of phenotype *i* per host (*N*_*i*_), the mean number of mf of phenotype *i* per milligram of skin per host (*M*_*i*_), and the mean number of infective (L3) larvae of phenotype *i* per blackfly vector (*L*_*i*_) in the population, *τ* time units following the administration of MDAi, where *i* ∈ {*GR, SOR*}.


(1) $$\frac{dN_{i}(t)}{dt}=\frac{1}{2}\beta m\Omega \Pi_{H}[L(t-p)]L_{i}(t-p)+\left(\lambda_{0}+v\lambda_{i}(\tau )\right)F_{i}(t)-\left(\omega_{0}-v\omega_{i}(\tau )+\sigma_{W}\right)N_{i}(t),$$

(2) $$\frac{dF_{i}(t)}{dt}=\left(\omega_{0}-v\;\omega_{i}(\tau )\right)N_{i}(t)-\left(\lambda_{0}+v\;\lambda_{i}(\tau )+\sigma_{W}\right)F_{i}(t),$$

(3) dMi(t)dt=εϕ[W(t)]WGR(t)W(t)WSOR(t)W(t)CiWSOR(t)W(t)ψGR(t)FGR(t)ψSOR(t)FSOR(t)-σM0+vσM1(τ)Mi(t),

(4) $$\frac{dL_{i}(t)}{dt}=\beta \Omega \Pi_{V}[M(t)]M_{i}(t)-\left(\frac{\alpha_{H}}{g}+\sigma_{L}+\mu_{V}+\alpha_{V}M(t)\right)L_{i}(t).$$

In Equations (1)–(4), *W*_*i*_(*t*) = *N*_*i*_(*t*) + *F*_*i*_(*t*) is the mean total number of adult female worms of phenotype *i* in a host, *W*(*t*) = *W*_*GR*_(*t*) + *W*_*SOR*_(*t*) is the mean total number of adult female worms in a host, *M*(*t*) = *M*_*GR*_(*t*) + *M*_*SOR*_(*t*) is the mean total number of mf per milligram of skin in a host, and *L*(*t*) = *L*_*GR*_(*t*) + *L*_*SOR*_(*t*) is the mean total number of infective larvae per blackfly. Variables are described in detail in Additional file 1.

A key difference between our model equations and those previously proposed is the inclusion of extra complexity in the terms describing the effects of ivermectin and rate of production of mf of phenotype *i* within human hosts, which is the result of sexual reproduction of worms with the same and different phenotypes. Below, we describe our approach to modelling the production of mf of each phenotype and the effect of phenotype on the response to ivermectin. All other model parameters are described fully in Table S1 of Additional file 1.

### Modelling the production of microfilariae and phenotype inheritance

The ivermectin response phenotype of *O. volvulus* is likely to be a complex genetic trait controlled by multiple loci, rather than a single locus [2], and is a spectrum of response phenotypes, ranging from an optimal response to ivermectin to no response [22]. Our model simplifies this continuous spectrum by considering two types of responses, GR and SOR, which is equivalent to defining a fertility threshold and classifying worms according to whether their fertility is above (SOR) or below (GR) this threshold following treatment (as in [2, 46, 48, 49].

Due to the likely complexity and uncertainty in the genetic mechanisms of inheritance of the response phenotypes, we take a phenomenological approach to modelling this process. As with previous models [52, 53], we assume polygamous mating between worms (i.e., a single male can fertilise all females within a host), so that the probability that a female worm mates with a male worm, Φ[*W*(*t*)], is approximated as the probability that at least one male worm is present in a host, assuming the worm distribution in hosts is negative binomial and an even sex ratio. We assume that because worms of each phenotype can be present in a single person [2], mating between phenotypes is possible and results in a proportion of new mf within the host with phenotype GR and the complementary proportion with phenotype SOR. The offspring phenotype matrix **C**_*GR*_[*x*(*t*)] has elements c_*i,j*_[*x*(*t*)] which represent the proportion of new mf of phenotype GR that result from a female worm of phenotype *j* ∈ {*GR, SOR*} mating with a male worm of phenotype *i* ∈ {*GR, SOR*}. The elements of the complementary proportion matrix **C**_*SOR*_[*x*(*t*)] are 1 – *c*_*i,j*_[*x*(*t*)]. We assume the matrices are symmetric (i.e., c_*i,j*_[*x*(*t*)] = c_*j,i*_[*x*(*t*)], *i* ≠ *j*). We also make the reasonable assumption that female worms can be fertilised by multiple male worms [13]. The matrices incorporate selection for the SOR phenotype following MDA through a piecewise linear dependence of *c*_*SOR,GR*_[*x*(*t*)] and *c*_*SOR,SOR*_[*x*(*t*)] on the average proportion *x*(*t*) = *W*_*SOR*_(*t*)/*W*(*t*) of worms in a host that are SOR at time *t*, which results in an increase in SOR mf and worm frequency after MDA. The selection coefficientss $s_{SOR,GR}=\frac{d}{dx}c_{SOR,GR}[x(t)]$ and $s_{SOR,SOR}=\frac{d}{dx}c_{SOR,GR}[x(t)]$ quantify the strength of selection for SOR during MDA programs.

With these assumptions, the rate of production of mf of phenotype *i* within human hosts is εϕ[W(t)]WGR(t)W(t)WSOR(t)W(t)CiWSOR(t)W(t)ψGR(t)FGR(t)ψSOR(t)FSOR(t). Here, *ε* is the natural rate of production of mf per fertile female worm, $\left(\frac{W_{GR}(t)}{W(t)}\frac{W_{\text{SOR}}(t)}{W(t)}\right)$ is a vector of the male worm phenotype probabilities and (*ψ*_*GR*_(*t*)*F*_*GR*_(*t*), *ψ*_*SOR*_(*t*)*F*_*SOR*_(*t*))^*T*^ is a vector of the mean fertile female worm loads, scaled by *ψ*_*i*_(*t*), the average reduction in the fertility of female worms of phenotype *i* due to the assumed cumulative effects of multiple exposures to ivermectin (discussed in more detail below).

### Effects of ivermectin

We adapt the approach described in Turner et al. [52] to model the microfilaricidal and embryostatic effects of ivermectin on GR and SOR worms. We assume the microfilaricidal effect on mf (which additively increases the death rate of mf by $\sigma_{M_{1}}(\tau )$, *τ* time units post the initiation of a new MDA round) is the same for both GR and SOR mf, but the embryostatic effects of ivermectin on worms (which we assume additively increases the rate at which fertile worms become infertile by *λ*_*i*_(*τ*) and decreases the rate at which non-fertile worms become fertile by *ω*_*i*_(*τ*), *τ* time units post the initiation of a new MDA round) decays slower in GR worms (at constant rate *φ*_*GR*_) compared to in SOR worms (at rate *φ*_*SOR*_ = *f φ*_*GR*_, where *f* > 1). We also include a mechanism in the model to account for the assumed level of the cumulative effect that may further suppress the fertility of female worms with additional exposures to ivermectin. This mechanism can reduce the rate of production of mf through the time-dependent functions *ψ*_*i*_(*t*) ∈ [0,1]. Given the uncertainty in whether or not this cumulative effect exists [54, 55, 56], we consider MDAi scenarios simulated with and without this cumulative effect on GR worms (we always assume SOR worms do not experience the cumulative effect, i.e., *ψ*_*SOR*_ ≡ 1 and *ψ*_*GR*_ ∈ [0,1]). The method used to calculate *ψ*_*GR*_(*t*) involves solving a system of delay differential equations tracking the total mean number of GR worms per host that have been exposed to $k\in N_{0}$ rounds of MDA, and is described in detail in Additional file 1: Text S1.

To model the effects of imperfect coverage of an MDA round, we scale the change in the transition rates between fertile and non-fertile worms, *ω*_*i*_(*τ*) and *λ*_*i*_(*τ*), as well as the increase in mf death rate, $\sigma_{M_{1}}(\tau )$, due to exposure to MDA by the proportion *v* of the population covered by the MDA. We also adjust the initial conditions of the delay differential equations used to calculate *ψ*_*GR*_(t) so that only the proportion *v* of the population covered by an MDA round increase their number of exposures to MDA by 1 unit (implicitly assuming that compliance is non-systematic).

### Effects of moxidectin

We assume that the microfilaricidal and embryostatic effects of moxidectin are mechanistically the same as those induced by ivermectin, but act at a higher level [33]. As MDAm is not currently applied at a large scale for onchocerciasis elimination, we do not know whether there exists a sub-optimally responsive phenotype to moxidectin. To assess the minimal relative benefit of MDAm compared to MDAi, we make the conservative assumption that it exists and has the same frequency as ivermectin SOR in treatment-naïve populations..

### Model parameterisation and calibration

The model is parameterised for transmission in savannah communities in Ghana or Cameroon where SOR female worms have been detected. All model parameters not related to the added complexity of multiple response phenotypes, with the exception of the per capita annual biting rate on humans from vectors, *ABR*, are fixed at point estimates based on previous work (described in detail in Additional file 1, Table S1). The *ABR* is calibrated for each transmission and treatment scenario to achieve the desired endemic level of mf prevalence in the population prior to MDAi. The approach used to simulate the model to reach endemic equilibrium in treatment naïve populations is described in Additional file 1 (Text S1).

Distributions for parameters related to the inheritance and effect of multiple response phenotypes in the model were determined by calibrating the model to be consistent with observations of mf prevalence and SOR worm frequency data in communities with and without histories of MDAi in Ghana, with, and then without, an assumed cumulative effect of ivermectin on female worm fertility. As these data do not exist for a single community, we use a combination of Latin Hypercube Sampling and a simple rejection sampling Approximate Bayesian Computation (ABC) method to identify model parameters consistent with these data. The use of this ABC method to establish consistency between a model and a set of beliefs (informed by, for example, empirical data, expert opinion, and/or theoretical considerations) is formalised in [57]. We defined the following three beliefs to calibrate our model: (1) the SOR worm proportion in MDAi naïve populations is between 0.05–0.15, in line with phenotype-based estimate of 0.12 from a treatment-naïve population in Ghana [2]; (2) when simulating a 20-year annual MDAi program with coverage levels matching those achieved in Asubende, Ghana between 1987–2006, the post-MDAi total mf prevalence was between 10–12%, in line with the observed mf prevalence of 11% in 2006, down from approximately 80% pre-MDAi in 1987 (Additional file 1: Figure S1, Table S1); and (3) the increase in mean SOR worm proportion in hosts due to the MDAi program was at least 0.1 (i.e., $\frac{W_{\text{SOR}}(20)}{W(20)}-\frac{W_{\text{SOR}}(0)}{W(0)}>0.1$), based on observed differences in the proportion of SOR worms between treatment-naïve and non-naïve populations in Cameroon of greater than 0.14 [2]. Given the uncertainty in the changes to the proportion of SOR worms that took place in Asubende due to MDAi, we performed a sensitivity analysis with a more conservative belief that the SOR worm proportion increase by 0.05–0.10 following the MDAi program. Further details of the model calibration process are provided in Additional file 1: Text S1.

### Analogous model without variable response to treatment

We also define an analogous model that does not account for variable response to treatment (instead, the model considers the transmission of GR parasites only) to allow us to make comparisons between (1) predictions of the impact of MDA programs in communities with SOR present from models that do, and do not, account for variable response to treatment; and (2) predictions of the impact of MDA programs in communities with and without the presence of SOR parasites. The GR-only model is constructed by setting *c*_*GR,GR*_ = 1 in equations (1)–(4) and specifying the initial conditions for the SOR model variables to be *N*_*SOR*_(0) = *F*_*SOR*_(0) = *M*_*SOR*_(0) = *L*_*SOR*_(0) = 0. We apply the same calibration approach for the GR-only model as we do with the variable response model (given an assumption that the response phenotype has no effect in treatment-naïve populations). The *ABR* is set at the same value in both models to achieve the same level of endemic mf prevalence. There is effectively only a single free parameter to identify when calibrating the GR-only model: the parameter *y*, which balances the embryostatic effect on transition rates between fertile and non-fertile female worms due to ivermectin.

### Transmission and MDA scenarios

We used the variable response model and the GR-only model to quantify differences in model predictions of the impact of MDAi programs on total mf prevalence in communities with and without SOR parasites. We explored how these predictions differ under varying levels of onchocerciasis endemicity (i.e., baseline mf prevalence) and under annual and biannual rounds of MDAi. In communities with SOR parasites present, the model with variable response phenotypes enabled us to quantify the impact of the MDAi programs on predicted changes to the frequency of SOR worms in communities under different frequencies of MDAi and under different assumptions about the level of onchocerciasis endemicity.

We then repeated our analyses to explore differences in model predictions under the theoretical impacts of MDAm programs compared to MDAi programs. We made the conservative assumption that the frequency of SOR to moxidectin occurs at the same baseline level in moxidectin-naïve populations as what has been observed for SOR to ivermectin in ivermectin-naïve populations (i.e., between 5–15%), and that the only difference between SOR and GR worms is their rate of loss of the embryostatic effects of moxidectin (which is estimated to be approximately 4.05 times slower than the analogous rates for ivermectin [39]). We also assume that both the increase in the rate at which fertile worms (of either genotype) become non-fertile, and the increase in the death rate of mf (of either genotype) due to moxidectin is faster than those rate increases for ivermectin (see Additional file 1: Table S1).

We implemented the model in R using the *dede* solver for delay differential equations available in the *deSolve* package [58]. All code used for all simulations reported in this manuscript are available at github.com/rhchisholm/two-strain-oncho-2026.

## Results

### Inclusion of variable response to ivermectin captures ongoing endemic transmission following annual and biannual MDAi programs in Asubende, Ghana.

No parameterization of the model, with or without variable response to ivermectin, fit the data from Asubende, Ghana (1987–2006) without incorporating a cumulative effect of additional exposures to ivermectin that further suppresses the fertility of female worms (Additional file 1: Figure S2). Therefore, this cumulative effect was assumed for all further simulations of both models.

Figure 2 shows the distribution of outcomes under the calibrated variable response model and the analogous calibrated model with the GR phenotype only. Following the 20-year annual MDAi program (run until the end of 2006), both models lead to almost identical predictions of the final mf prevalence. This is expected as both models were calibrated to the final mf prevalence (11%) observed in Asubende after the 20-year annual MDAi program. However, there is a significant difference between the predictions of the models following the subsequent 18-year biannual MDAi program. The GR-only model predicted that elimination was possible under the biannual MDAi program with a final mf prevalence of 0.51%, which is below the Provisional Operational Thresholds for Treatment Interruption and commencement of Surveillance (pOTTIS) of 1.4% (Basáñez et al., 2016). This was not the case under the variable response model, which predicted a median final mf prevalence of 4.6% (95% CI: [3.0, 5.3]) (Figure 2, Additional file 1: Table S2), and consistent with observations of ongoing transmission in Asubende following the annual and biannual MDAi programs between 1987–2024.

### The frequency of SOR phenotype increases during periods of annual and biannual rounds of MDAi

An advantage of the variable response model is that it enables the theoretical exploration of likely changes to the frequency of SOR worms compared to GR worms in communities under MDAi programs. In the modelled Asubende scenario described above, the 20-year annual MDAi program led to a theoretical increase in mean SOR worm proportion (i.e., $\frac{W_{SOR}(t)}{W(t)}$) from a median of 0.11 (95% CI: [0.08, 0.14]) up to a median of 0.22 (95% CI: [0.19, 0.26]) (Additional file 1: Figure S4A, Table S2). The subsequent 18-year biannual MDAi program led to a further increase in SOR worm proportion up to median of 0.49 (95% CI: [0.46, 0.52]) (Additional file 1: Figure S4B, Table S2). The increase in SOR worm proportion was higher due to the biannual MDAi than it was during the annual MDAi program. Note, however, that these time periods covered different starting frequencies of SOR, different program durations, and MDAi coverages. In the next section, we keep these parameters fixed to understand how the frequency of MDAi and baseline endemic mf prevalence affect the rate of increase of SOR due to MDAi in treated communities.

### Effects of MDAi programs in meso- and hyperendemic communities with and without SOR

The Asubende scenario allowed us to explore the effects of MDAi programs in a community hyperendemic for onchocerciasis and with SOR present (with a starting mf prevalence of 80%). Next, we explored whether predicted effects of MDAi programs differed in meso- and hyperendemic communities (specifically, pre-MDAi mf prevalence of 30%, 50% and 70%) given variable frequency and duration of MDAi (20 rounds of annual MDAi versus 20 or 40 rounds of biannual MDAi), and when the MDAi programs were run in communities either with or without the presence of SOR. In all scenarios, the distributions for the unknown model parameters were based on the accepted parameter sets from calibrating the variable response model to the Asubende data (i.e., those summarised in Additional file 1: Figure S3), and the population coverage of each MDAi round was assumed to be 80%.

**Figure 3.**
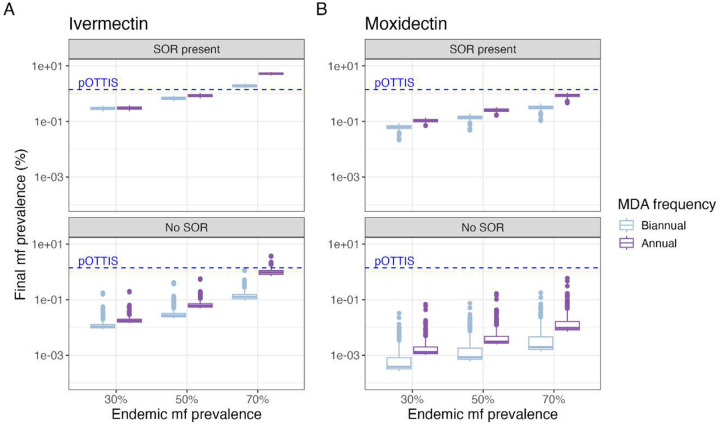
Predictions of mf prevalence following MDA programs for different levels of baseline onchocerciasis endemic transmission, with and without the presence of SOR worms at baseline. The boxplots summarise the distributions for the final mf prevalence following (purple) 20-year annual; and (blue) 10-year biannual (A) MDAi; and (B) MDAm, programs in communities that had 30%, 50% or 70% mf prevalence at baseline, with either (top row) SOR and GR worms present at baseline; or (bottom row) only GR present at baseline. The horizontal dashed blue line is positioned where the final mf prevalence equals pOTTIS.

**Figure 4.**
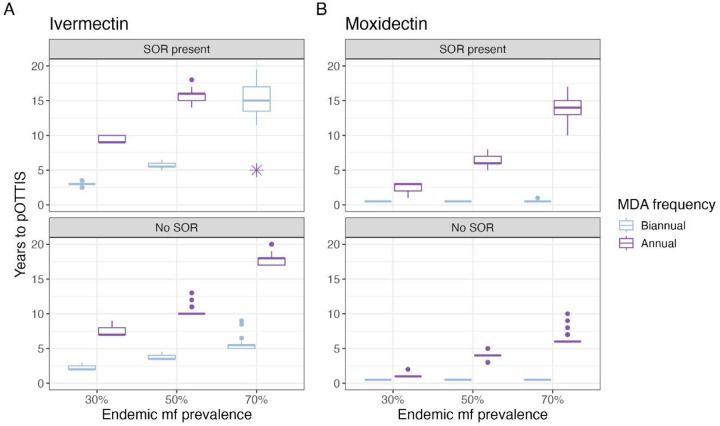
Predictions of time taken to reach pOTTIS by implementing MDA programs for different levels of baseline onchocerciasis endemic transmission, with and without the presence of SOR worms at baseline. The boxplots summarise the distributions for the time in years to reach pOTTIS following (purple) annual; and (blue) biannual (A) MDAi; and (B) MDAm, programs in communities that had 30%, 50% or 70% mf prevalence at baseline, with either (top row) SOR and GR worms present at baseline; or (bottom row) only GR present at baseline. Simulations were run for a maximum of 20 years, so box plots represent time to pOTTIS conditional on pOTTIS being reached within 20 years. Some simulations of MDAi scenarios with 70% mf prevalence at baseline did not reach pOTTIS within 20 years. Specifically, pOTTIS was not reached under any accepted parameter combinations within 20 years for the annual MDAi program with 70% mf prevalence at baseline when SOR was present in the population (indicated by the *), and under 11% of simulations in the corresponding scenario without SOR.

**Figure 5.**
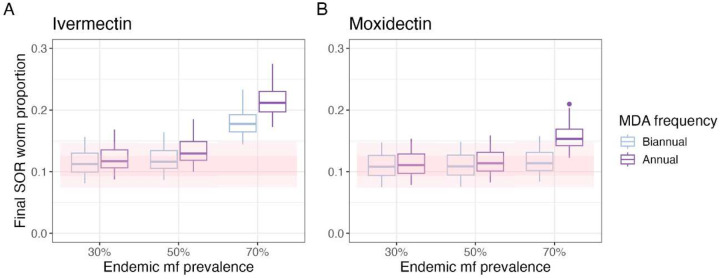
Predictions of final SOR worm proportion following 20-year MDA programs under the variable response model for different levels of baseline onchocerciasis endemic transmission. The boxplots summarise the distributions for the final SOR worm proportion under the variable response model following (purple) 20-year annual; and (blue) 20-year biannual (A) MDAi; and (B) MDAm, programs in communities that had 30%, 50% or 70% mf prevalence at baseline. The pink bands represent the distributions of the initial SOR worm proportion prior to MDA under each scenario, with the darker bands corresponding to the interquartile ranges, and the lighter bands to all values within the distributions, excluding outliers.

The results for all baseline endemic transmission and MDAi scenarios are summarised in Figure 3–5 and Additional file 1: Tables S3-S11. For both 20 rounds of annual and biannual MDAi, and for all baseline starting prevalence values, the variable response model predicted a higher final mf prevalence post-MDAi program in communities with SOR present compared to analogous predictions under the GR-only model in communities without SOR (Figure 3A). The relative difference between predictions of the final mf prevalence in communities with and without SOR was greater for biannual MDAi compared to annual MDAi, and for lower levels of baseline endemic mf prevalence. For example, when the baseline endemic mf prevalence was set at 50%, the final mf prevalence post annual MDAi program in the variable response model with SOR present was a median of 0.85% (95% CI: [0.70, 1.02]%) compared to 0.06% (95% CI: [0.05, 0.17]%) in the corresponding GR-only scenario (i.e., a 13.98 times higher median final mf prevalence when SOR is present), while after the biannual MDAi program it was a median of 0.68% (95% CI: [0.56, 0.83]%) compared to 0.03% (95% CI: [0.02, 0.12]%) in the corresponding GR-only scenario (i.e., a 25.26 times higher median final mf prevalence when SOR is present). When the baseline endemic mf prevalence was set at 70%, the median final mf prevalence post-annual MDAi program in the variable response model with SOR present was above pOTTIS at 5.22% (95% CI: [4.60, 5.87]%) compared to being below pOTTIS in the GR-only scenario at 0.96% (95% CI: [0.71, 1.82]%). This was also the case after the biannual MDAi program; the variable response model predicted a median of 1.90% (95% CI: [1.60, 2.28]%) compared to 0.13% (95% CI: [0.09, 0.44]%) in the corresponding GR-only scenario. For these higher baseline mf prevalence scenarios, the relative median final mf prevalences between communities with and without SOR were lower compared to the 50% baseline scenarios, at 5.44 and 14.92, respectively.

When the baseline level of endemic mf prevalence was set at either 30% or 50%, the models indicate that communities with and without SOR parasites under annual and biannual MDAi programs could achieve pOTTIS within 20 years. In these scenarios, the duration of the MDAi programs required to reach pOTTIS was shorter for biannual MDAi compared to annual MDAi, and shorter for 30% baseline mf prevalence scenarios compared to the corresponding 50% baseline mf prevalence scenarios (Figure 4A). For example, in communities with 50% baseline endemic mf prevalence, the time to reach pOTTIS under annual MDAi programs in the variable response model with SOR present was a median of 16 (95% CI: [14, 17]) years compared to 10 (95% CI: [10, 11]) years in the corresponding GR-only scenario, while after the biannual MDAi program it was a median of 5.5 (95% CI: [5, 6.5]) years compared to 3.5 (95% CI: [3.5, 4]) years in the corresponding GR-only scenario. When the baseline endemic mf prevalence was set at 30%, the median time to reach pOTTIS with annual MDAi under the variable response model with SOR present was 9 (95% CI: [9, 10]) years compared to 7 (95% CI: [7, 8]) years in the corresponding GR-only scenario. When the baseline level of endemic mf prevalence was set to 70%, pOTTIS could not be reached within 20 years in communities undergoing annual MDAi with SOR parasites present under any accepted parameter combinations of the variable reponse model. In the corresponding simulations of the GR-only model, 11% of simulations did not reach pOTTIS within 20 years.

The frequency of MDAi and baseline level of endemic mf prevalence also affected the predicted changes to the frequency of SOR worms compared to GR worms in communities with SOR present under the variable response model (Figure 5A). Larger increases in mean SOR worm proportion from a median of 0.11 (95% CI: [0.08, 0.15]) occurred following 20 years of annual MDAi compared to 20 years of biannual MDAi. For example, when the baseline endemic mf prevalence was set at 70%, the final mean SOR worm proportion following the 20-year annual MDAi program was a median of 0.21 (95% CI: [0.18, 0.26]), while following the 20-year biannual MDAi program it was a median of 0.18 (95% CI: [0.15, 0.21]). When the baseline endemic mf prevalence was set at either 30% or 50%, minimal changes occurred to the distributions of final mean SOR worm proportion due to biannual MDAi programs, and some slight increases occurred to these distributions due to annual MDAi programs (Additional file 1: Figure 5A, Table S11).

### Effects of MDAm programs in meso- and hyperendemic communities with and without SOR

We repeated our analyses to explore the differences in model predictions under the theoretical impacts of MDAm programs compared to MDAi programs. The results for all MDAm scenarios are summarised in Figures 3–5 and Additional file 1: Table S5-S12. For both annual and biannual MDAm programs and in communities with and without SOR parasites present, our models predicted a lower final mf prevalence post-MDAm program compared to the analogous MDAi programs, for all levels of baseline endemic transmission considered (Figure 3). In all MDAm scenarios, the relative difference in predictions of final mf prevalence between communities with and without SOR were larger compared to the equivalent MDAi scenarios. For example, when the baseline endemic mf prevalence was set at 70%, the annual MDAm program led to an 87.5 times higher median prediction of final mf prevalence in communities with SOR compared to those without SOR.

The effects of the frequency of MDAm and the baseline level of endemic mf prevalence on model predictions were comparable to those observed under MDAi programs. For example, when the baseline endemic mf prevalence was set at 70% under the variable response model, biannual MDAm led to a final median mf prevalence of 0.32% (95% CI: [0.17, 0.41%]) and after annual MDAm it was a median of 0.88% (95% CI: [0.62, 1.05%]), i.e., a median that was 2.70 times higher. Under the analogous MDAi scenarios, annual MDAi led to a median final mf prevalence that was 2.76 times higher than biannual MDAi. In contrast to MDAi, however, reaching pOTTIS was predicted to be possible under all MDAm programs considered, even in the scenarios with a baseline endemic mf prevalence of 70% (Figure 3–4).

When we contrast the theoretical changes to the frequency of SOR worms compared to GR worms in communities under MDAi versus MDAm programs, we found that MDAm programs led to lower frequencies of SOR worms under all endemic transmission and MDA frequency scenarios considered (Figure 5, Additional file 1: Tables S11-S12). For example, when the baseline endemic mf prevalence was set at 70%, the final SOR worm proportion post annual MDAm program was a median of 0.15 (95% CI: [0.13, 0.19]), while after the annual MDAi program it was a median of 0.21 (95% CI: [0.18, 0.26]).

### Sensitivity analysis

Given the limited data on the effect of selection from MDAi programs on SOR worm frequency, we performed a sensitivity analysis using the variable response model but calibrated to a smaller expected increase in SOR worm proportion following the 20-year annual MDAi program in Asubende (Figure S2). The results are summarised in Additional file 1: Figures S6-S10, Tables S13–23. Under this parameterisation, the predicted reduction of mf prevalence of both MDAi and MDAm programs is higher relative to predictions under models assuming stronger selection for SOR worms (compare to Figure 2–3 and Additional file 1: Figure S4, Figures S6-S8). However, the qualitative findings detailed above do not change under this model parameterisation.

## Discussion

We present here an epidemiological model that incorporates within-population variation in parasite response to ivermectin, the drug currently used in MDA for onchocerciasis. While our model treats this variation as a binary trait, and does not explicitly incorporate the genetic basis for this trait, it represents a key first step for predicting the impacts of stop-MDA decisions when there is variation in parasite drug response. Empirical data from initial and subsequent studies on the effect of MDAi on parasite prevalence and on worm fertility indicates that individual *O. volvulus* vary in their phenotypic response to ivermectin exposure, and that this variation exists within populations [2, 22, 42, 43, 46, 47, 48, 49]. The epidemiological modelling presented here supports concerns raised in the research community that SOR to ivermectin in *O. volvulus* could result in persistent transmission despite high-coverage, biannual MDAi in communities with high baseline endemicity.

### Cumulative impact of ivermectin on female worm fertility

Assessments of the long-term impacts of multiple rounds of ivermectin on the fertility of female worms is complicated by the inability to track mf production by individual females over time—*O. volvulus* has a reproductive cycle that only occurs within the human host, and examination of individual females requires surgical removal of entire nodules [12, 15, 46, 48, 49, 59, 60, 61, 62]. Statistical studies examining the burden of mf in individual people have suggested that there is a cumulative effect of ivermectin on female worms, based on the observation that the rate of mf skin repopulation is lower than would be expected solely by aging and death of female worms [54, 55, 63, 64]. While this has been widely accepted within the onchocerciasis community, at least one report indicates that this effect may vary across parasite populations [56].

The results of our data-fitting indicated that even in an area with long-term ivermectin use and persistent transmission (Asubende, Ghana), incorporating a cumulative effect of ivermectin on fertility of female worms better fit the data than with no cumulative effect. Previous modelling indicates that this cumulative effect decreases the duration of annual or biannual MDAi required to reach elimination of transmission [53], results that are echoed by the modelling performed here when there are only GR worms in the population. In this scenario—cumulative effect, minimum treatment coverage of 80%, no variable worm response to ivermectin—MDAi would effectively eliminate transmission in Asubende given a sufficient number of rounds of treatment (Fig 2).

### Selection for SOR worms

In scenarios where we assume SOR parasites are present, over successive rounds of MDA, regardless of the baseline endemicity, the proportion of worms that are SOR is predicted to increase due to selection under our variable response model. At baseline, when the worm population is ivermectin naïve, we have assumed that a certain proportion of worms already meet the binary SOR threshold [65], and that the naïve worm population is at mutation-selection equilibrium: there is no selection for or against genetic variation that would affect drug response, and the rate of new genetic mutations that would contribute to the SOR phenotype would be equal to the rate of loss of other SOR-contributing mutations. Thus, in our variable response model, the frequency of SOR worms remains constant over time in the absence of MDA.

MDA results in selection for genetic variation that contributes to SOR and thus increases the proportion of SOR worms over time. This increase in proportion is higher when there is annual rather than biannual MDA: the longer that SOR mf have to populate the skin of infected people, the greater advantage SOR worms have for being transmitted, and will represent a higher proportion in the next generation of reproducing worms.

### Effects of SOR on elimination

After a single round of treatment, mf gradually repopulate the skin as females recover fertility. When there is annual compared to biannual MDAi, there are mf in the skin for a longer proportion of time, correspondingly higher transmission, and thus a higher prevalence after 20 rounds of annual MDAi compared to the same number of rounds of biannual MDAi (Fig 3). When there are only GR worms, biannual ivermectin is predicted to clear most mf prior to skin repopulation, reducing the number of mf available for blackflies to take up and transmit to another person. Consistent with previous modelling results [53] that assume there are only GR worms, elimination (i.e., reaching the elimination threshold, pOTTIS) is possible even in a baseline hyperendemic area, such as Asubende, with high annual blackfly biting rates (i.e., baseline mf prevalence ≥70%). When there are SOR worms, however, reaching the elimination threshold in hyperendemic areas is predicted to take 2–3 times as many rounds of MDAi.

Many meso- and hypoendemic communites were not included in MDAi campaigns when they were begun. Assuming an initial starting percentage of SOR worms of only ~10%, these communities are expected to reach elimination thresholds even with the presence of SOR worms at the start of MDAi, and are not predicted to require additional rounds to do so (Fig 4).

### Will moxidectin solve the SOR problem?

Data from clinical trials suggest that moxidectin is a promising alternative to ivermectin for MDA [21, 22, 23], and modelling performed here and elsewhere has indicated that fewer rounds of moxidectin would be required to achieve effective elimination of transmission [20, 41]. There are no data suggesting that SOR for moxidectin exists in *O. volvulus*. However, variation in response to moxidectin has been demonstrated in other nematodes [66, 67, 68], and thus we have explored how the benefits of MDAm might be challenged if there is a cognate response similar to that observed with MDAi. If we assume similar underlying mechanisms (i.e., selection for female fertility as a multi-locus, heritable trait), variation in MDAm response in *O. volvulus* would lead to selection for, and an increase in the proportion of, worms with SOR to moxidectin over time. However, even with this selective pressure, our model does not predict that these worms would significantly increase the rounds of MDA needed to reach elimination of transmission when compared to a model with only GR to moxidectin (Figs 3,4).

### Future directions for model improvement

Movement of infected people between communities, whether for work, to escape civil conflicts, or other reasons, or movement of infective blackflies, could contribute to persistent transmission between communities [69, 70, 71, 72]. The model presented here assumes no connectivity with other communities. Future incorporation of the impact of community connectivity on increasing the risk of SOR spreading between endemic areas, or, conversely, on reducing the risk of persistent transmission from SOR because of dilution by GR worms, is recommended.

We further do not treat variation in drug response as a continuous trait in the female worm population, although it most certainly is [42, 46, 48]. An important next step would be to incorporate SOR mathematically as a continuous (quantitative) trait whose distribution changes over time and/or specifically incorporating population genetic-based processes. Towards this end, a more comprehensive understanding of the underlying genetics behind SOR would be helpful, particularly since preliminary research in this area suggests that loci involved in SOR differs between geographic locations [2]. Unfortunately, collecting and sequencing sufficient numbers of individual post-ivermectin treatment phenotyped adult female worms or mf for robust genome-wide association studies has proven to be challenging [16, 73].

A final assumption is that SOR predominantly impacts female worms. However, ivermectin may be impacting the behaviour of male worms by disrupting their ability to migrate to nodules containing fertile females [74]. A male-driven sub-optimal response, in which SOR males are either not disrupted by ivermectin or are able to recover more quickly than GR males, would impact female fertility because of the availability of sperm rather than changes in female recovery times. Examination of whether alleles associated with early mf development *in utero* within (presumed) SOR female worms are associated with fertilization by a particular father would be extremely valuable in determining whether male SOR should be included in epidemiological models.

### Conclusion

Ivermectin has been an extremely effective tool for control of onchocerciasis, and has resulted in successful elimination in countries across the globe [75]. Monitoring, surveillance, and decision-making strategies in areas where onchocerciasis persists need take into account the complex mosaic of communities connected by migratory behaviour of blackflies and of people, geographic and seasonal changes in blackfly densities, and parasite evolution in response to MDA. The modelling presented here supports the need to acknowledge the potential problems of SOR when making decisions about how to optimally achieve elimination where efforts have stalled. Unfortunately, current protocols for identifying changes in the proportion of SOR worms are laborious, requiring surgical extraction of nodules followed by embryogram quantification of offspring development, or by following up with individual people as soon as three months post-ivermectin treatment to estimate mf prevalence [42, 43]. A DNA-based, rapid test to identify the proportion of SOR worms in a community, combined with modelling that accounts for variation in drug response, would allow programmes to estimate the risk of SOR to elimination and to adapt strategies dynamically, either by incorporating alternative drugs such as moxidectin or antibiotics and/or targeted reduction of blackfly breeding habitats or larviciding.

## Supplementary Material

Supplementary Files

This is a list of supplementary files associated with this preprint. Click to download.

- ChisholmetalSORAdditionalfile110Jun2026v2.docx

Additional file 1:

**Text S1.** Supplementary methods describing the variable response model without the effects of MDAi, further details about the calibration method and results for the variable response and GR-only model.

**Figure S1.** Calibrating the variable response model; and GR-only model, to mf prevalence data from Asubende following a 20-year annual MDAi program.

**Figure S2.** Final distributions of accepted parameter samples for the variable response model.

**Figure S3.** Final distributions for the final model mf prevalence and final increase in SOR worm proportion after annual and biannual MDAi programs in Asubende using the variable response model parameterised with the accepted parameter samples.

**Figure S4.** Predictions of final mf prevalence following 10-year biannual and 20-year annual MDAi and MDAm programs under the variable response model for different levels of baseline onchocerciasis endemic transmission.

**Table S1.** Definitions and values of model parameters.

**Table S2.** Statistics of the marginal posterior distributions for the unknown model parameters and corresponding distributions for the simulation summary statistics following model calibration to Asubende annual MDAi data.

**Table S3.** Summary statistics of the distributions of the relative final mf prevalence post MDAi program in the variable-response model compared to the GR-only model for different frequencies of MDAi rounds and levels of endemic mf prevalence at baseline prior to MDAi.

**Table S4.** Summary statistics of the distributions of the final mf prevalence post MDAi program in the variable-response model for different frequencies of MDAi rounds and levels of endemic mf prevalence at baseline prior to MDAi.

**Table S5.** Summary statistics of the distributions of the relative final mf prevalence post MDAm program in the variable-response model compared to the GR-only model for different frequencies of MDAm rounds and levels of endemic mf prevalence at baseline prior to MDAm.

**Table S6.** Summary statistics of the distributions of the final mf prevalence post MDAm program in the variable-response model for different frequencies of MDAm rounds and levels of endemic mf prevalence at baseline prior to MDAm.

**Table S7.** Summary statistics of the distributions of the final SOR worm proportion post MDAi program in the variable-response model for different frequencies of MDAi rounds and levels of endemic mf prevalence at baseline prior to MDAi.

**Table S8.** Summary statistics of the distributions of the final SOR worm proportion post MDAm program in the variable-response model for different frequencies of MDAm rounds and levels of endemic mf prevalence at baseline prior to MDAm.

## Figures and Tables

**Figure 1.**
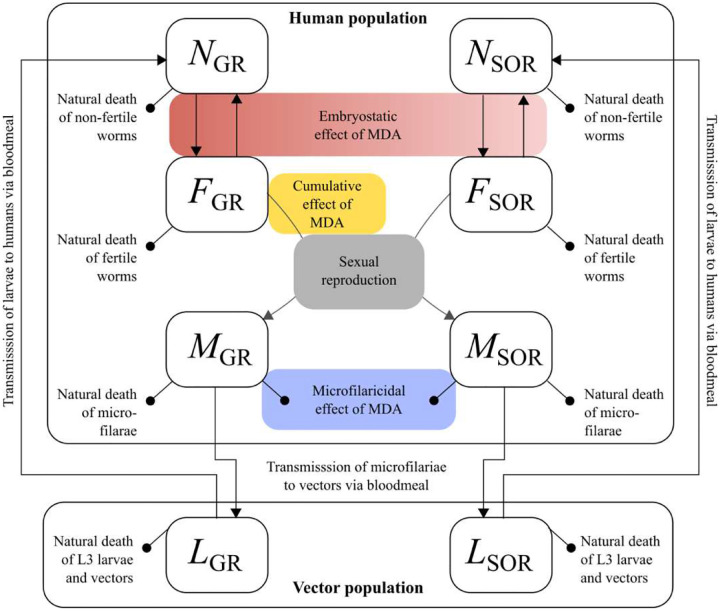
Schematic diagram of the two-phenotype *Onchocerca volvulus* transmission model with mass drug administration. Model states are shown as unshaded boxes and model state transitions are shown as arrows. Model state transitions that are affected by mass drug administration (MDA) are shaded red for the embryostatic effect, yellow for the cumulative effect on fertility, blue for the microfilaricidal effect, and grey for selection for sub-optimal response (SOR). Model states include *N*_*GR*_, non-fertile good responding (GR) female worms in human hosts; *F*_*GR*_, fertile GR female worms in human hosts; *M*_*GR*_, microfilariae that will be GR adults in human hosts; *N*_*SOR*_, non-fertile SOR female worms in human hosts; *F*_*SOR*_, fertile SOR female worms in human hosts; *M*_*SOR*_, microfilariae that will be SOR adults in human hosts; *L*_*GR*_, L3 larvae in vectors that will be GR adults; *L*_*SOR*_, L3 larvae in vectors that will be SOR adults; MDA, mass drug administration.

## Data Availability

All R code used to produce simulation results is available at [github.com/rhchisholm/two-strain-oncho-2026].

## Abbreviations

GR: good-responding worms
SOR: sub-optimal responding worms
mf: microfilariae
pOTTIS: *p*rovisional *o*perational *t*hreshold for *t*reatment *i*nterruption and commencement of *s*urveillance [53]
MDAi: mass drug administration with ivermectin
MDAm: mass drug administration with moxidectin

## References

[1] Bourguinat C, Lee AC, Lizundia R, Blagburn BL, Liotta JL, Kraus MS, Macrocyclic lactone resistance in *Dirofilaria immitis*: Failure of heartworm preventives and investigation of genetic markers for resistance. Vet Parasitol. 2015;210 3–4:167–78; doi: 10.1016/j.vetpar.2015.04.002. http://www.ncbi.nlm.nih.gov/pubmed/25936435. 25936435

[2] Doyle SR, Bourguinat C, Nana-Djeunga HC, Kengne-Ouafo JA, Pion SDS, Bopda J, Genome-wide analysis of ivermectin response by *Onchocerca volvulus* reveals that genetic drift and soft selective sweeps contribute to loss of drug sensitivity. PLoS Negl Trop Dis. 2017;11 7:e0005816; doi: 10.1371/journal.pntd.0005816. http://www.ncbi.nlm.nih.gov/pubmed/28746337. 28746337 PMC5546710

[3] Baltrusis P, Doyle SR, Halvarsson P, Hoglund J. Genome-wide analysis of the response to ivermectin treatment by a Swedish field population of Haemonchus contortus. Int J Parasitol Drugs Drug Resist. 2022;18:12–9; doi: 10.1016/j.ijpddr.2021.12.002. https://www.ncbi.nlm.nih.gov/pubmed/34959200. 34959200 PMC8718930

[4] Choi YJ, Bisset SA, Doyle SR, Hallsworth-Pepin K, Martin J, Grant WN, Genomic introgression mapping of field-derived multiple-anthelmintic resistance in Teladorsagia circumcincta. PLoS Genet. 2017;13 6:e1006857; doi: 10.1371/journal.pgen.1006857. http://www.ncbi.nlm.nih.gov/pubmed/28644839. 28644839 PMC5507320

[5] Grant WN. Genetic variation in parasitic nematodes and its implications. Int J Parasitol. 1994;24 6:821–30. http://www.ncbi.nlm.nih.gov/pubmed/7982744. 7982744 10.1016/0020-7519(94)90008-6

[6] Hedtke SM, Kuesel AC, Crawford KE, Graves PM, Boussinesq M, Lau CL, Genomic epidemiology in filarial nematodes: transforming the basis for elimination program decisions. Front Genet. 2020;10:1282; doi: 10.3389/fgene.2019.01282. https://www.ncbi.nlm.nih.gov/pubmed/31998356. 31998356 PMC6964045

[7] Coffeng LE, Stolk WA, de Vlas SJ. Predicting the risk and speed of drug resistance emerging in soil-transmitted helminths during preventive chemotherapy. Nat Commun. 2024;15 1:1099; doi: 10.1038/s41467-024-45027-2. https://www.ncbi.nlm.nih.gov/pubmed/38321011. 38321011 PMC10847116

[8] World Health Organization. Elimination of human onchocerciasis: progress report, 2022–2023 – Élimination de l’onchocercose humaine: rapport de situation, 2022–2023. Weekly Epidemiological Record. 2023;98 45:572–82. https://iris.who.int/handle/10665/373991.

[9] Brattig NW. Pathogenesis and host responses in human onchocerciasis: impact of Onchocerca filariae and Wolbachia endobacteria. Microbes and Infection. 2004;6 1:113–28; doi: 10.1016/j.micinf.2003.11.003. https://www.sciencedirect.com/science/article/pii/S1286457903003101. 14738900

[10] Chesnais CB, Nana-Djeunga HC, Njamnshi AK, Lenou-Nanga CG, Boulle C, Bissek AZ, The temporal relationship between onchocerciasis and epilepsy: a population-based cohort study. Lancet Infect Dis. 2018;18 11:1278–86; doi: 10.1016/S1473-3099(18)30425-0. http://www.ncbi.nlm.nih.gov/pubmed/30268645. 30268645

[11] Schulz-Key H, Albiez EJ. Worm burden of *Onchocerca volvulus* in a hyperendemic village of the rain-forest in West Africa. Tropenmedizin und Parasitologie. 1977;28 4:431–8. 601852

[12] Duke BO, Zea-Flores G, Gannon RT. On the reproductive activity of the female *Onchocerca volvulus*. Trop Med Parasitol. 1990;41 4:387–402. http://www.ncbi.nlm.nih.gov/pubmed/2075383. 2075383

[13] Kumar P, Choi YJ, Fischer K, Hedtke SM, Kode A, Opoku N, Genetic structuring and estimation of reproductive adults in *Onchocerca volvulus*: A genome-wide analysis across hosts and regions. PLoS Negl Trop Dis. 2025;19 7:e0013221; doi: 10.1371/journal.pntd.0013221. https://www.ncbi.nlm.nih.gov/pubmed/40591654. 40591654 PMC12212510

[14] Duke BO. The population dynamics of *Onchocerca volvulus* in the human host. Trop Med Parasitol. 1993;44 2:61–8. http://www.ncbi.nlm.nih.gov/pubmed/8367667. 8367667

[15] Albiez EJ, Büttner DW, Schulz-Key H. Studies on nodules and adult *Onchocerca volvulus* during a nodulectomy trial in hyperendemic villages in Liberia and Upper Volta. II. Comparison of the macrofilaria population in adult nodule carriers. Tropenmed Parasitol. 1984;35:163–6. 6541822

[16] Hedtke SM, Choi Y-J, Kode A, Chalasani GC, Sirwani N, Jada SR, Assessing intensity of infection and genetic diversity of onchocerciasis using mitochondrial genome sequencing of single microfilariae. Pathogens. 2023;12 7:971; doi: 10.3390/pathogens12070971. 37513818 PMC10385737

[17] World Health Organization, African Programme for Onchocerciasis Control: Conceptual and operational framework of onchocerciasis elimination with ivermectin treatment. vol. WHO/APOC/MG/10.1. Burkina Faso: World Health Organization; 2010.

[18] World Health Organization: Guidelines for stopping mass drug administration and verifying elimination of human onchocerciasis: criteria and procedures. Geneva: World Health Organization; 2016. 26913317

[19] Awadzi K, Dadzie KY, Schulz-Key H, Haddock DR, Gilles HM, Aziz MA. Ivermectin in onchocerciasis. Lancet. 1984;2 8408:921. http://www.ncbi.nlm.nih.gov/pubmed/6148634. 10.1016/s0140-6736(84)90671-8 6148634

[20] Turner HC, Walker M, Attah SK, Opoku NO, Awadzi K, Kuesel AC, The potential impact of moxidectin on onchocerciasis elimination in Africa: an economic evaluation based on the Phase II clinical trial data. Parasit Vectors. 2015;8:167; doi: 10.1186/s13071-015-0779-4. http://www.ncbi.nlm.nih.gov/pubmed/25889256. 25889256 PMC4381491

[21] Opoku NO, Bakajika DK, Kanza EM, Howard H, Mambandu GL, Nyathirombo A, Single dose moxidectin versus ivermectin for *Onchocerca volvulus* infection in Ghana, Liberia, and the Democratic Republic of the Congo: a randomised, controlled, double-blind phase 3 trial. Lancet. 2018;392 10154:1207–16; doi: 10.1016/S0140-6736(17)32844-1. http://www.ncbi.nlm.nih.gov/pubmed/29361335. 29361335 PMC6172290

[22] Bakajika D, Kanza EM, Opoku NO, Howard HM, Mambandu GL, Nyathirombo A, Effect of a single dose of 8 mg moxidectin or 150 μg/kg ivermectin on *O. volvulus* skin microfilariae in a randomized trial: Differences between areas in the Democratic Republic of the Congo, Liberia and Ghana and impact of intensity of infection. PLoS Negl Trop Dis. 2022;16 4:e0010079; doi: 10.1371/journal.pntd.0010079. https://www.ncbi.nlm.nih.gov/pubmed/35476631. 35476631 PMC9084535

[23] Awadzi K, Opoku NO, Attah SK, Lazdins-Helds J, Kuesel AC. A randomized, single-ascending-dose, ivermectin-controlled, double-blind study of moxidectin in *Onchocerca volvulus* infection. PLoS Negl Trop Dis. 2014;8 6:e2953; doi: 10.1371/journal.pntd.0002953. http://www.ncbi.nlm.nih.gov/pubmed/24968000. 24968000 PMC4072596

[24] Routledge I, Walker M, Cheke RA, Bhatt S, Nkot PB, Matthews GA, Modelling the impact of larviciding on the population dynamics and biting rates of Simulium damnosum (s.l.): implications for vector control as a complementary strategy for onchocerciasis elimination in Africa. Parasites & Vectors. 2018;11 1:316; doi: 10.1186/s13071-018-2864-y. https://doi.org/10.1186/s13071-018-2864-y. 29843770 PMC5972405

[25] Tan B, Opoku N, Attah SK, Awadzi K, Kuesel AC, Lazdins-Helds J, Pharmacokinetics of oral moxidectin in individuals with Onchocerca volvulus infection. PLOS Neglected Tropical Diseases. 2022;16 3:e0010005; doi: 10.1371/journal.pntd.0010005. https://doi.org/10.1371/journal.pntd.0010005. 35333880 PMC8986118

[26] Opoku NO, Doe F, Agbogah ME, Laryea R, Gordor SK, Donkor BS, Identification of a moxidectin dose for 4- to 11-year-old children to support registration and potential use for onchocerciasis elimination: results of an open-label pharmacokinetic and safety study. Parasit Vectors. 2025;18 1:295; doi: 10.1186/s13071-025-06891-z. https://www.ncbi.nlm.nih.gov/pubmed/40707999. 40707999 PMC12291498

[27] Plaisier AP, van Oortmarssen GJ, Habbema JD, Remme J, Alley ES. ONCHOSIM: a model and computer simulation program for the transmission and control of onchocerciasis. Comput Methods Programs Biomed. 1990;31 1:43–56. http://www.ncbi.nlm.nih.gov/pubmed/2311368. 2311368 10.1016/0169-2607(90)90030-d

[28] Hamley JID, Milton P, Walker M, Basáñez MG. Modelling exposure heterogeneity and density dependence in onchocerciasis using a novel individual-based transmission model, EPIONCHO-IBM: Implications for elimination and data needs. PLoS Negl Trop Dis. 2019;13 12:e0007557; doi: 10.1371/journal.pntd.0007557. https://www.ncbi.nlm.nih.gov/pubmed/31805049. 31805049 PMC7006940

[29] Basáñez MG, Boussinesq M. Population biology of human onchocerciasis. Philos Trans R Soc Lond B Biol Sci. 1999;354 1384:809–26; doi: 10.1098/rstb.1999.0433. http://www.ncbi.nlm.nih.gov/pubmed/10365406. 10365406 PMC1692549

[30] Coffeng LE, Stolk WA, Hoerauf A, Habbema D, Bakker R, Hopkins AD, Elimination of African onchocerciasis: modeling the impact of increasing the frequency of ivermectin mass treatment. PLoS One. 2014;9 12:e115886; doi: 10.1371/journal.pone.0115886. http://www.ncbi.nlm.nih.gov/pubmed/25545677. 25545677 PMC4278850

[31] Stolk WA, Walker M, Coffeng LE, Basáñez MG, de Vlas SJ. Required duration of mass ivermectin treatment for onchocerciasis elimination in Africa: a comparative modelling analysis. Parasit Vectors. 2015;8:552; doi: 10.1186/s13071-015-1159-9. http://www.ncbi.nlm.nih.gov/pubmed/26489937. 26489937 PMC4618738

[32] Basáñez MG, Ricardez-Esquinca J. Models for the population biology and control of human onchocerciasis. Trends Parasitol. 2001;17 9:430–8. http://www.ncbi.nlm.nih.gov/pubmed/11530355. 11530355 10.1016/s1471-4922(01)02013-x

[33] Basáñez MG, Walker M, Turner HC, Coffeng LE, de Vlas SJ, Stolk WA. River blindness: mathematical models for control and elimination. Adv Parasitol. 2016;94:247–341; doi: 10.1016/bs.apar.2016.08.003. http://www.ncbi.nlm.nih.gov/pubmed/27756456. 27756456

[34] Verver S, Walker M, Kim YE, Fobi G, Tekle AH, Zoure HGM, How can onchocerciasis elimination in Africa be accelerated? Modeling the impact of increased ivermectin treatment frequency and complementary vector control. Clin Infect Dis. 2018;66 suppl_4:S267–S74; doi: 10.1093/cid/cix1137. http://www.ncbi.nlm.nih.gov/pubmed/29860291. 29860291 PMC5982715

[35] Stolk WA, Blok DJ, Hamley JID, Cantey PT, de Vlas SJ, Walker M, Scaling-down mass ivermectin treatment for onchocerciasis elimination: modeling the impact of the geographical unit for decision making. Clin Infect Dis. 2021;72 Suppl 3:S165–S71; doi: 10.1093/cid/ciab238. https://www.ncbi.nlm.nih.gov/pubmed/33909070. 33909070 PMC8201558

[36] de Vos AS, Stolk WA, Coffeng LE, de Vlas SJ. The impact of mass drug administration expansion to low onchocerciasis prevalence settings in case of connected villages. PLoS Negl Trop Dis. 2021;15 5:e0009011; doi: 10.1371/journal.pntd.0009011. https://www.ncbi.nlm.nih.gov/pubmed/33979331. 33979331 PMC8143415

[37] Filipe JA, Boussinesq M, Renz A, Collins RC, Vivas-Martinez S, Grillet ME, Human infection patterns and heterogeneous exposure in river blindness. Proc Natl Acad Sci U S A. 2005;102 42:15265–70; doi: 10.1073/pnas.0502659102. https://www.ncbi.nlm.nih.gov/pubmed/16217028. 16217028 PMC1257694

[38] de Vos AS, Stolk WA, de Vlas SJ, Coffeng LE. The effect of assortative mixing on stability of low helminth transmission levels and on the impact of mass drug administration: Model explorations for onchocerciasis. PLoS Negl Trop Dis. 2018;12 10:e0006624; doi: 10.1371/journal.pntd.0006624. https://www.ncbi.nlm.nih.gov/pubmed/30296264. 30296264 PMC6175282

[39] Turner HC, Churcher TS, Walker M, Osei-Atweneboana MY, Prichard RK, Basáñez MG. Uncertainty surrounding projections of the long-term impact of ivermectin treatment on human onchocerciasis. PLoS Negl Trop Dis. 2013;7 4:e2169; doi: 10.1371/journal.pntd.0002169. http://www.ncbi.nlm.nih.gov/pubmed/23634234. 23634234 PMC3636241

[40] Vinkeles Melchers NVS, Stolk WA, Murdoch ME, Pedrique B, Kloek M, Bakker R, How does onchocerciasis-related skin and eye disease in Africa depend on cumulative exposure to infection and mass treatment? PLoS Negl Trop Dis. 2021;15 6:e0009489; doi: 10.1371/journal.pntd.0009489. https://www.ncbi.nlm.nih.gov/pubmed/34115752. 34115752 PMC8221783

[41] Kura K, Milton P, Hamley JID, Walker M, Bakajika DK, Kanza EM, Can mass drug administration of moxidectin accelerate onchocerciasis elimination in Africa? Philos Trans R Soc Lond B Biol Sci. 2023;378 1887:20220277; doi: 10.1098/rstb.2022.0277. https://www.ncbi.nlm.nih.gov/pubmed/37598705. 37598705 PMC10440165

[42] Awadzi K, Attah SK, Addy ET, Opoku NO, Quartey BT, Lazdins-Helds JK, Thirty-month follow-up of sub-optimal responders to multiple treatments with ivermectin, in two onchocerciasis-endemic foci in Ghana. Ann Trop Med Parasitol. 2004;98 4:359–70; doi: 10.1179/000349804225003442. http://www.ncbi.nlm.nih.gov/pubmed/15228717. 15228717

[43] Awadzi K, Boakye DA, Edwards G, Opoku NO, Attah SK, Osei-Atweneboana MY, An investigation of persistent microfilaridermias despite multiple treatments with ivermectin, in two onchocerciasis-endemic foci in Ghana. Ann Trop Med Parasitol. 2004;98 3:231–49; doi: 10.1179/000349804225003253. http://www.ncbi.nlm.nih.gov/pubmed/15119969. 15119969

[44] Osei-Atweneboana MY, Eng JK, Boakye DA, Gyapong JO, Prichard RK. Prevalence and intensity of *Onchocerca volvulus* infection and efficacy of ivermectin in endemic communities in Ghana: a two-phase epidemiological study. Lancet. 2007;369 9578:2021–9; doi: 10.1016/S0140-6736(07)60942-8. http://www.ncbi.nlm.nih.gov/pubmed/17574093. 17574093

[45] Churcher TS, Pion SD, Osei-Atweneboana MY, Prichard RK, Awadzi K, Boussinesq M, Identifying sub-optimal responses to ivermectin in the treatment of River Blindness. Proc Natl Acad Sci U S A. 2009;106 39:16716–21; doi: 10.1073/pnas.0906176106. http://www.ncbi.nlm.nih.gov/pubmed/19805362. 19805362 PMC2757820

[46] Osei-Atweneboana MY, Awadzi K, Attah SK, Boakye DA, Gyapong JO, Prichard RK. Phenotypic evidence of emerging ivermectin resistance in *Onchocerca volvulus*. PLoS Negl Trop Dis. 2011;5 3:e998; doi: 10.1371/journal.pntd.0000998. http://www.ncbi.nlm.nih.gov/pubmed/21468315. 21468315 PMC3066159

[47] Pion SD, Nana-Djeunga HC, Kamgno J, Tendongfor N, Wanji S, Njiokou F, Dynamics of Onchocerca volvulus microfilarial densities after ivermectin treatment in an ivermectin-naive and a multiply treated population from Cameroon. PLoS Negl Trop Dis. 2013;7 2:e2084; doi: 10.1371/journal.pntd.0002084. http://www.ncbi.nlm.nih.gov/pubmed/23469307. 23469307 PMC3585010

[48] Nana-Djeunga HC, Bourguinat C, Pion SD, Bopda J, Kengne-Ouafo JA, Njiokou F, Reproductive status of *Onchocerca volvulus* after ivermectin treatment in an ivermectin-naive and a frequently treated population from Cameroon. PLoS Negl Trop Dis. 2014;8 4:e2824; doi: 10.1371/journal.pntd.0002824. http://www.ncbi.nlm.nih.gov/pubmed/24762816. 24762816 PMC3998936

[49] Frempong KK, Walker M, Cheke RA, Tetevi EJ, Gyan ET, Owusu EO, Does increasing treatment frequency address suboptimal responses to ivermectin for the control and elimination of River blindness? Clin Infect Dis. 2016;62 11:1338–47; doi: 10.1093/cid/ciw144. http://www.ncbi.nlm.nih.gov/pubmed/27001801. 27001801 PMC4872292

[50] Abong RA, Amambo GN, Chounna Ndongmo PW, Njouendou AJ, Ritter M, Beng AA, Differential susceptibility of *Onchocerca volvulus* microfilaria to ivermectin in two areas of contrasting history of mass drug administration in Cameroon: relevance of microscopy and molecular techniques for the monitoring of skin microfilarial repopulation within six months of direct observed treatment. BMC Infect Dis. 2020;20 1:726; doi: 10.1186/s12879-020-05444-2. https://www.ncbi.nlm.nih.gov/pubmed/33008333. 33008333 PMC7530974

[51] Turner HC, Walker M, Churcher TS, Basáñez MG. Modelling the impact of ivermectin on River blindness and its burden of morbidity and mortality in African savannah: EpiOncho projections. Parasit Vectors. 2014;7:241; doi: 10.1186/1756-3305-7-241. http://www.ncbi.nlm.nih.gov/pubmed/24886747. 24886747 PMC4037555

[52] Turner HC, Osei-Atweneboana MY, Walker M, Tettevi EJ, Churcher TS, Asiedu O, The cost of annual versus biannual community-directed treatment of onchocerciasis with ivermectin: Ghana as a case study. PLoS Negl Trop Dis. 2013;7 9:e2452; doi: 10.1371/journal.pntd.0002452. http://www.ncbi.nlm.nih.gov/pubmed/24069497. 24069497 PMC3777881

[53] Turner HC, Walker M, Churcher TS, Osei-Atweneboana MY, Biritwum NK, Hopkins A, Reaching the London Declaration on Neglected Tropical Diseases goals for onchocerciasis: an economic evaluation of increasing the frequency of ivermectin treatment in Africa. Clin Infect Dis. 2014;59 7:923–32; doi: 10.1093/cid/ciu467. http://www.ncbi.nlm.nih.gov/pubmed/24944228. 24944228 PMC4166981

[54] Plaisier AP, Alley ES, Boatin BA, Van Oortmarssen GJ, Remme H, De Vlas SJ, Irreversible effects of ivermectin on adult parasites in onchocerciasis patients in the Onchocerciasis Control Programme in West Africa. J Infect Dis. 1995;172 1:204–10. http://www.ncbi.nlm.nih.gov/pubmed/7797912. 7797912 10.1093/infdis/172.1.204

[55] Basáñez M-G, Pion SD, Boakes E, Filipe JA, Churcher TS, Boussinesq M. Effect of single-dose ivermectin on *Onchocerca volvulus*: a systematic review and meta-analysis. Lancet Infect Dis. 2008;8 5:310–22; doi: 10.1016/S1473-3099(08)70099-9. http://www.ncbi.nlm.nih.gov/pubmed/18471776. 18471776

[56] Bottomley C, Isham V, Collins RC, Basáñez MG. Rates of microfilarial production by *Onchocerca volvulus* are not cumulatively reduced by multiple ivermectin treatments. Parasitology. 2008;135 13:1571–81; doi: 10.1017/S0031182008000425. https://www.ncbi.nlm.nih.gov/pubmed/18831801. 18831801

[57] Vollert SA, Drovandi C, Jeyes-Smith C, Pascal LV, Adams MP. Beyond data: leveraging non-empirical information and expert knowledge in Bayesian model calibration. arXiv. 2025:arXiv:2505:21934; doi: 10.48550/arXiv.2505.21934.

[58] Soetaert K, Petzoldt T, Setzer RW. Solving Differential Equations in R. R J. 2010;2 2:5–15. <Go to ISI>://WOS:000208590000002.

[59] Schulz-Key H. The collagenase technique: how to isolate and examine adult *Onchocerca volvulus* for the evaluation of drug effects. Trop Med Parasitol. 1988;39 Suppl 4:423–40. http://www.ncbi.nlm.nih.gov/pubmed/2852394. 2852394

[60] Schulz-Key H. Observations on the reproductive biology of *Onchocerca volvulus*. Acta Leiden. 1990;59 1–2:27–44. https://www.ncbi.nlm.nih.gov/pubmed/2378210. 2378210

[61] Schulz-Key H, Albiez EJ, Buttner DW. Isolation of living adult *Onchocerca volvulus* from nodules. Tropenmed Parasitol. 1977;28 4:428–30. 203061

[62] Schulz-Key H, Karam M. Periodic reproduction of *Onchocerca volvulus*. Parasitol Today. 1986;2 10:284–6. http://www.ncbi.nlm.nih.gov/pubmed/15462735. 15462735 10.1016/0169-4758(86)90138-9

[63] Gardon J, Boussinesq M, Kamgno J, Gardon-Wendel N, Demanga N, Duke BO. Effects of standard and high doses of ivermectin on adult worms of *Onchocerca volvulus*: a randomised controlled trial. Lancet. 2002;360 9328:203–10; doi: 10.1016/S0140-6736(02)09456-4. https://www.ncbi.nlm.nih.gov/pubmed/12133654. 12133654

[64] Cupp EW, Cupp MS. Short report: impact of ivermectin community-level treatments on elimination of adult *Onchocerca volvulus* when individuals receive multiple treatments per year. Am J Trop Med Hyg. 2005;73 6:1159–61. http://www.ncbi.nlm.nih.gov/pubmed/16354830. 16354830

[65] !!! INVALID CITATION !!! [2].

[66] Molento MB, Wang GT, Prichard RK. Decreased ivermectin and moxidectin sensitivity in *Haemonchus contortus* selected with moxidectin over 14 generations. Vet Parasitol. 1999;86 1:77–81. http://www.ncbi.nlm.nih.gov/pubmed/10489206. 10489206 10.1016/s0304-4017(99)00131-4

[67] Blackhall WJ, Liu HY, Xu M, Prichard RK, Beech RN. Selection at a P-glycoprotein gene in ivermectin- and moxidectin-selected strains of *Haemonchus contortus*. Mol Biochem Parasitol. 1998;95 2:193–201. http://www.ncbi.nlm.nih.gov/pubmed/9803412. 9803412 10.1016/s0166-6851(98)00087-5

[68] Menez C, Alberich M, Kansoh D, Blanchard A, Lespine A. Acquired tolerance to ivermectin and moxidectin after drug selection pressure in the nematode *Caenorhabditis elegans*. Antimicrob Agents Chemother. 2016;60 8:4809–19; doi: 10.1128/AAC.00713-16. http://www.ncbi.nlm.nih.gov/pubmed/27246778. 27246778 PMC4958191

[69] Hedtke SM, Post RJ, Feleke SM, Gebretsadik FS, Boakye DA, Krueger A, Cytotaxonomic characterization and estimation of migration patterns of onchocerciasis vectors (*Simulium damnosum sensu lato*) in northwestern Ethiopia based on RADSeq data. PLoS Negl Trop Dis. 2024;18 1:e0011868; doi: 10.1371/journal.pntd.0011868. 38175836 PMC10793886

[70] Koala L, Nikiema AS, Pare AB, Drabo F, Toe LD, Belem AMG, Entomological assessment of the transmission following recrudescence of onchocerciasis in the Comoé Valley, Burkina Faso. Parasit Vectors. 2019;12 1:34; doi: 10.1186/s13071-019-3290-5. http://www.ncbi.nlm.nih.gov/pubmed/30646934. 30646934 PMC6332526

[71] Nikièma AS, Koala L, Post RJ, Paré AB, Kafando CM, Drabo F, Onchocerciasis prevalence, human migration and risks for onchocerciasis elimination in the Upper Mouhoun, Nakambe and Nazinon river basins in Burkina Faso. Acta Trop. 2018;185:176–82; doi: 10.1016/j.actatropica.2018.05.013. http://www.ncbi.nlm.nih.gov/pubmed/29782820. 29782820

[72] Shrestha H, McCulloch K, Chisholm RH, Armoo S, Vierigh F, Sirwani N, Synthesizing environmental, epidemiological, and genetic data to understand the persistence of onchocerciasis transmission in the ecological transition region of Ghana. Molec Ecol. 2024;33 1:e17357; doi: 10.1111/mec.17357. 38683054

[73] Hedtke SM, Kode A, Ukety TO, Mande JL, Abhafule GM, Raciu AA, Procedure for handling and storage of skin snips for downstream genetic work on *Onchocerca volvulus* microfilariae. Trop Med Infect Dis. 2023;8 9:445; doi: 10.3390/tropicalmed8090445. https://www.mdpi.com/2414-6366/8/9/445. 37755906 PMC10536066

[74] Lok JB, Knight DH, Selavka CM, Eynard J, Zhang Y, Bergman RN. Studies of reproductive competence in male *Dirofilaria immitis* treated with milbemycin oxime. Trop Med Parasitol. 1995;46 4:235–40. http://www.ncbi.nlm.nih.gov/pubmed/8826103. 8826103

[75] World Health Organization = Organisation mondiale de la Santé. Elimination of human onchocerciasis: progress report, 2024–2025 = Élimination de l’onchocercose humaine: rapport de situation, 2024–2025. Weekly Epidemiological Record. 2025;100 41:451–60. https://iris.who.int/handle/10665/383100.

